# Bibliometric Study of Trends in the Diabetic Nephropathy Research Space from 2016 to 2020

**DOI:** 10.1155/2022/8050137

**Published:** 2022-04-12

**Authors:** Ying Shao

**Affiliations:** Department of Endocrinology, Shengjing Hospital of China Medical University, Shenyang, Liaoning, China

## Abstract

**Background:**

Diabetic nephropathy (DN) is one of the most common microvascular complications of diabetes mellitus (DM), but no bibliometric studies pertaining to DN have been published within the last 5 years.

**Objectives:**

Most prior studies have focused on specific problems in the DN field. This study attempts to sort out and visualize the knowledge framework in this research space from a holistic and highly generalized perspective. Readers can quickly understand and master the knowledge regarding DN research conducted from 2016 to 2020, in addition to predicting future research hotspots and possible directions for development in this field in a comprehensive and scientifically valid manner.

**Methods:**

Literature information, discourse matrices, and co-occurrence matrices were generated using BICOMB. gCLUTO was used for biclustering analyses and visualization. Strategic diagrams were generated using GraphPad Prism 5. The social network analysis (SNA) was analyzed and plotted using Ucinet 6.0 and Netdraw.

**Results:**

In total, 55 high-frequency MeSH terms/MeSH subheadings were selected and grouped into 5 clusters in a biclustering analysis. These analyses revealed that extensive studies of the etiology, diagnosis, and treatment of DN have been conducted over the last 5 years, while further research regarding DN-related single nucleotide polymorphisms, miRNAs, and signal transduction are warranted as these research areas remain relatively immature.

**Conclusion:**

Together, these results outline a robust knowledge structure pertaining to the field of DN-related research over the last 5 years, providing a valuable resource for readers by enabling the easy comprehension of relevant information. In addition, this analysis highlights predicted DN-related research directions and hotspots.

## 1. Introduction

Diabetic nephropathy (DN) is among the most debilitating complications of type 2 diabetes mellitus (T2DM). Given that DN progresses to end-stage renal disease (ESRD) in an estimated 45% of patients [1], DN has emerged as one of the leading causes of ESRD at the global level. While rates of many other diabetes-related complications have declined over the past three decades, the same has not held true for DN [2]. The pathogenesis of DN is characterized by basement membrane thickening and glomerular extracellular matrix accumulation [3], with proteinuria serving as one of the earliest clinical signs of this disease prior to its progression to ESRD [4]. To date, traditional diabetes-related therapies such as weight loss, glycemic control, and renin-angiotensin-aldosterone system (RAAS) inhibitor administration have failed to yield satisfactory clinical outcomes as a means of treating DN [5].

Many studies pertaining to the diagnosis, treatment, molecular pathogenesis, etiology, and epidemiology of DN have emerged in recent years. Major breakthroughs over the past 5 years have included the identification of sodium-glucose cotransporter 2 (SGLT2) inhibitors as tools for DN treatment [6] and the value of specific microRNAs (miRNAs) as biomarkers for DN patient diagnosis [7]. These important research findings have led to the publication of a wealth of research, yet no bibliometric analyses of DN-related knowledge structure or research trends have been published to date based on studies from the last 5 years included in the PubMed database.

PubMed represents one of the most comprehensive and authoritative sources of scientific literature. Medical bibliometry [8, 9] has emerged as an approach to compiling, analyzing, and presenting high-level overviews of research pertaining to a particular topic in specific databases using appropriate software tools, enabling researchers to assess extant research in a quantitative manner while clarifying the overall structure of the field and identifying promising research hotspots. Co-word analyses are the most common bibliometric approach, with biclustering analyses in which rows and columns of a given matrix are simultaneously clustered for subsequent visualization also frequently being employed. The results of these biclustering analyses can then be visualized based upon their centrality and density in the form of a strategic diagram that is reflective of current research status. Moreover, a social network analysis (SNA) can be performed to more appropriately visualize network relationships among high-frequency topics and terms in a particular research space, thereby enabling the visualization of possible research hotspots.

In summary, most previous studies in the DN research space have focused on specific issues such as the treatment of DN, diagnosis of DN, or etiology of DN. Herein, we sought to conduct a bibliometric analysis of data compiled from the PubMed database in order to gain insight into the status of research within the DN field over the past 5 years. In addition, this study is committed to providing a comprehensive and informative overview of this topic suited with visual tools to guide biomedical researchers, medical educators, medical students, and clinicians.

## 2. Data and Methods

### 2.1. Data Collection

All studies included in the present analysis were retrieved and downloaded from the National Center for Biotechnology Information PubMed database. Studies in this database are indexed and classified based on MeSH terms. For this analysis, the retrieval term was “Diabetic Nephropathies” [MeSH], and there were no specific language limitations nor were species restricted during the retrieval process. As the goal of this study was to assess the overall structure of DN research over the past 5 years and associated trends, only studies published from 1 January 2016 to 31 December 2020 were retrieved. To avoid any potential outcome biases due to updates to the PubMed database, the retrieval and downloading of all 5,337 studies in the present analysis were completed on 14 October 2021. Journal titles, country of origin, publication date, and associated MeSH terms/MeSH subheadings were saved in the XML format. This study was approved by the Ethics Institutional Review Board of China Medical University.

### 2.2. Study-Related Matrix Generation and Data Extraction

Data extraction and analysis for the 5,337 studies retrieved from the PubMed database were performed using a BICOMB (Bibliographic Item Co-Occurrence Matrix Builder) algorithm [10]. Terms-publication and co-occurrence matrices were then generated, and these served as raw data for subsequent statistical analyses. This BICOMB approach was used to characterize a variety of parameters pertaining to retrieved studies including countries of origin, journals, and key MeSH terms/MeSH subheadings.

MeSH terms and subheadings that were present at a high frequency were identified via the H-index method. Briefly, these MeSH terms and subheadings were initially ranked in descending frequency order, after which those terms and subheadings with a word frequency greater than or equal to the corresponding serial number (H) were selected such that H was the overall threshold for MeSH term/MeSH subheading inclusion in downstream analyses.

### 2.3. Biclustering Analysis of High-Frequency MeSH Terms/MeSH Subheadings

PubMed Unique Identifier (PMID) and high-frequency MeSH terms/subheadings for DN-related studies identified in the PubMed database were extracted and analyzed with BICOMB, with the resultant terms-publication matrix being utilized to conduct a biclustering analysis. A visual peak diagram in which each cluster is represented by a three-dimensional (3D) topographical peak was generated by analyzing this matrix using the gCLUTO software. In the resultant diagram, peak height is proportional to the similarity within the cluster, while the distance between peaks corresponds to the similarity between clusters, and peak volume is proportional to the number of objects within that cluster. The coloration of the apex of the peak is determined based on the intracluster variation, with a scale from red to blue, respectively, corresponding to values ranging from small to large. Column labels in the generated biclustering diagram correspond to study PMIDs, while row labels correspond to high-frequency MeSH terms/MeSH subheadings. Dendrograms and corresponding clustering are shown with this biclustering diagram, and all clusters were summarized and analyzed based on the results of this biclustering process.

### 2.4. Strategic Diagram Analysis

Strategic diagrams are two-dimensional (2D) graphs that enable the visualization of research trends and overall knowledge structure in a given field, with the *x*-axis and *y*-axis of these diagrams, respectively, corresponding to centrality and density [11]. In this analysis, centrality is defined as the strength of the connection between a particular theme and other topics, while density is defined as the strength of associations between basic knowledge units within a given subject. Greater density values thus correspond to a higher degree of cluster maturity. For this study, a strategic diagram was generated using data from biclustering analyses, with centrality and density for individual clusters being calculated as reported previously [12]. Coordinates were drawn using GraphPad Prism 5 (GraphPad Software Inc., CA, USA).

### 2.5. Social Network Analysis

The co-occurrence matrix of high-frequency MeSH terms/MeSH subheadings extracted from BICOMB was used by the Ucinet software to calculate parameters including degree centrality, betweenness centrality, and closeness centrality. The results of these analyses were then used to conduct a social network analysis (SNA) aimed at clarifying relationships among high-frequency MeSH terms/MeSH subheadings for the purposes of data quantification and visualization. In the SNA plot generated for the present analysis, nodes corresponded to 55 high-frequency MeSH terms/MeSH subheadings of interest, while the thickness of lines between these nodes was proportional to the co-occurrence frequency of these different pairs of MeSH terms/subheadings. Degree centrality corresponds to the number of connections between nodes, offering some insight into the overall value of a given node within the network. Betweenness centrality corresponds to the number of shortest paths between all node pairs, providing insight regarding the load capacity of a given node, thus making this a core network indicator. Node size was thus proportional to betweenness centrality. Closeness centrality corresponds to the reciprocal of the shortest distance between a node and all other reachable nodes with normalization following accumulation.

**Figure 1:**
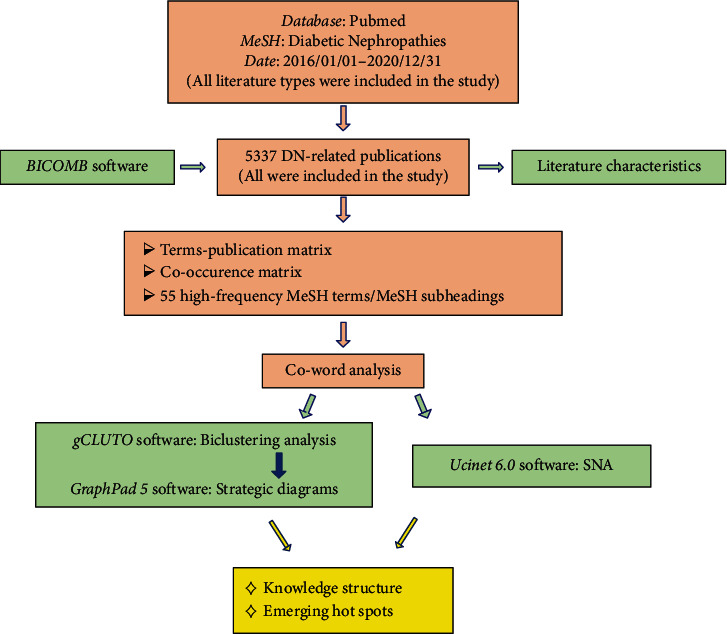
Research design flow chart. BICOMB: Bibliographic Item Co-Occurrence Matrix Builder; MeSH: Medical Subjects Headings; SNA: social network analysis.

A research design flow chart is provided in Figure 1.

## 3. Results

### 3.1. Characteristics of DN-Related Literature

Using the search strategy outline above, 5,337 DN-related studies published from 2016 to 2020 were identified. The top 20 journal names and corresponding countries associated with these studies are compiled in Table 1. The USA was the most published nation (33.37%), followed by the UK and the Netherlands. In total, DN-related studies were published in 905 journals, among which the most frequent were the *Journal of Diabetes Research* (IF: 4.011, 2020), *Scientific Reports* (IF: 4.379, 2020), and the *Journal of Diabetes and Its Complications* (IF: 2.852, 2020), accounting for 8.35% of the overall research output in this study.

### 3.2. Identification of DN Research Hotspots through a Biclustering Analysis of MeSH Terms and Subheadings

**Figure 2:**
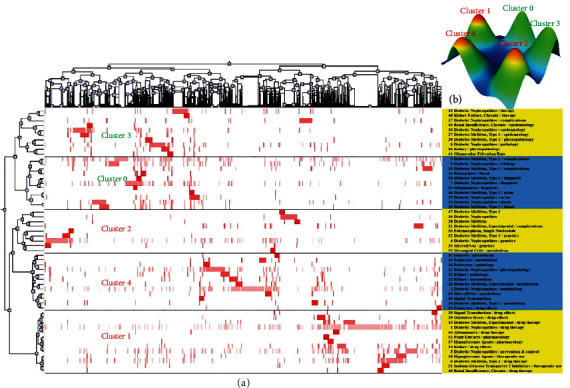
Biclustering analysis of 55 high-frequency MeSH terms/MeSH subheadings and publications pertaining to DN from 2016 to 2020. (a) Matrix visualization of biclustering results for 55 high-frequency MeSH terms/MeSH subheadings with corresponding publication PMIDs. (b) Mountain visualization of the biclustering analysis results.

Next, the 55 highest-frequency MeSH terms/MeSH subheadings were extracted from these 5,337 DN-related studies, with a cumulative frequency of 37.4737%. These terms were considered to correspond to hotspots in the DN research space over the past 5 years. These terms were grouped into 5 clusters in a biclustering analysis (Figure 2 and Table 2). Specifically, Figure 2 includes the individual MeSH terms and subheadings within each cluster on the right, while the corresponding dendrograms display the relationships between these terms and the corresponding literature. Table 2 displays the contents of each cluster and the results of corresponding analyses.

### 3.3. Strategic Diagram-Based Overview of the DN Research Space

We next generated a strategic diagram based on the results of biclustering analyses of high-frequency MeSH terms and MeSH subheadings associated with DN-related research published over the last 5 years in order to better visualize connections within and between different topics and to more fully quantify the developmental maturity of different research topics within this field. In the generated diagram, the *x*-axis corresponds to the core degree value, which is indicative of the importance of a given cluster, while the *y*-axis corresponds to maturity, indicating the degree of development within a given cluster. This model yields four distinct clusters, with quadrant I in the upper right corresponding to topics that exhibit both a high core degree and relative maturity consistent with an important and relatively well-studied topic. In contrast, quadrant II in the upper left exhibits a low core degree but a high degree of maturity consistent with topics that may be less important but are nonetheless very maturely developed within the field. Quadrant III in the lower left corresponds to research that exhibits a lower core degree and a low degree of maturity, suggesting that these topics may correspond to emerging areas with the potential for further intensive research. Lastly, quadrant IV in the lower right corresponds to topics that exhibit a high core degree but lower maturity, suggesting that while these research areas may be highly important, they remain incompletely developed and likely warrant further investigation. Overall, strategic diagrams provide a representation of the structure of a given research field over a defined time frame, with clusters exhibiting time-dependent mobility. As the topics included in clusters III and IV exhibit relatively low levels of research maturity, they may represent optimal areas for new research with the potential to emerge as hotspots for further research.

**Figure 3:**
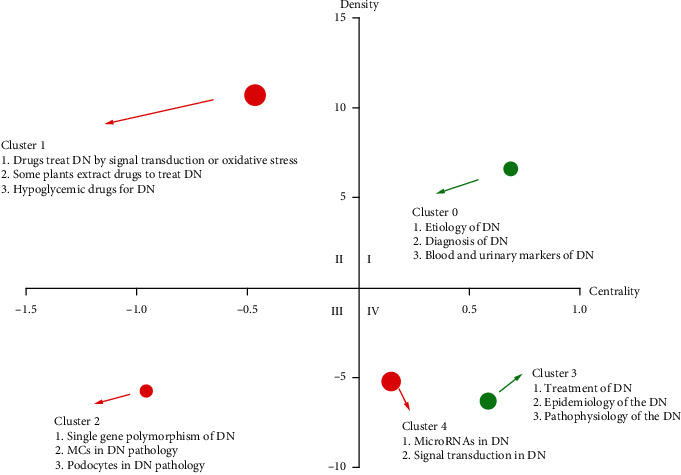
Strategic diagram pertaining to DN-related research published from 2016 to 2020. Node sizes correspond to the number of MeSH terms/MeSH subheadings associated with each cluster.

Here, we generated a strategic diagram corresponding to the results of our biclustering analysis of 55 high-frequency MeSH terms and subheadings pertaining to identified DN-related studies published over the last 5 years. In this analysis, cluster 0 was located in quadrant I and was associated with keywords including Etiology, Diagnosis, and Blood and urinary markers, suggesting that this cluster primarily corresponds to the Etiology and Diagnosis of DN, with studies in which urine- and blood-based biomarkers were used to guide patient diagnosis being mature and recognized as important. Cluster 1 was located in quadrant II and associated with keywords including drugs and treatment, with a particular focus on drugs with an antioxidant mechanism of action, plant-derived drugs, and drugs used to treat hypoglycemia. Given that the drug-related treatment of DN has been a focus of ongoing research for many years, this cluster exhibited a high degree of maturity but a slightly lower core degree in the present analysis. Cluster 2 was located in quadrant III and was associated with keywords pertaining to the roles of monogenic polymorphisms, mesangial cells, and podocytes in the pathogenesis of DN. As this clustered exhibited lower core degree and maturity values, it may represent an area with extensive opportunities for future research. Cluster 3 was associated with keywords pertaining to the epidemiology and pathophysiology of DN, while cluster 4 was associated with keywords including DN-related miRNAs and signal transduction pathways. Both of these clusters were located within quadrant IV, consistent with a high core degree but relatively limited maturity at present (Figure 3).

### 3.4. Social Network Analysis

**Figure 4:**
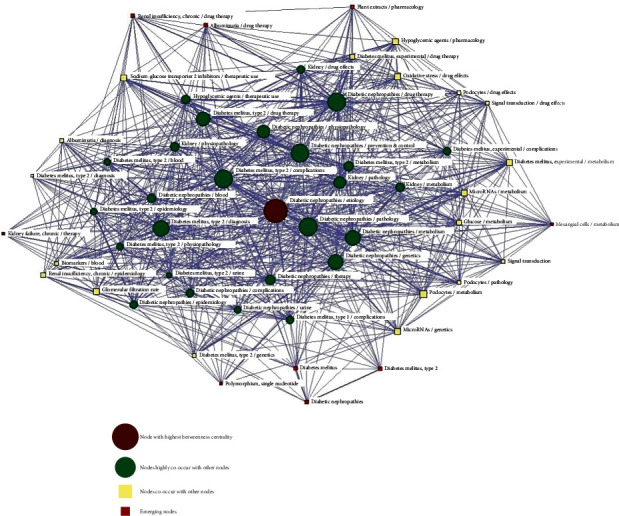
Social network analysis (SNA) for 55 high-frequency MeSH terms/MeSH subheadings pertaining to DN-related studies published from 2016 to 2020. Nodes correspond to MeSH terms/MeSH subheadings, with node size and location being indicative of word centrality within the SNA. Edges correspond to connections between terms, with line thickness corresponding to the co-occurrence frequency of the indicated MeSH terms/MeSH subheading pairs.

To better visualize the relationships among the identified high-frequency MeSH terms/MeSH subheadings associated with the DN-related research space over the past 5 years, we next generated an SNA diagram (Figure 4). For this analysis, three individual matrices corresponding to degree centrality, betweenness centrality, and closeness centrality were generated by extracting the co-occurrence matrix generated above and inputting it into Ucinet. The thickness of lines in the resultant diagram was proportional to the co-occurrence frequency for the corresponding pairs of terms.

The top three betweenness centrality values in this SNA were 46.170, 35.649, and 35.636 (Table 3), respectively, corresponding to the following MeSH term/subheading pairs: Diabetic Nephropathies/etiology, Diabetic Nephropathies/drug therapy, and Diabetes Mellitus, Type 2/complications. These terms thus function as key bridges within the overall complex network. The top two MeSH terms/subheadings with respect to degree centrality were Diabetic Nephropathies/drug therapy (1167) and Diabetes Mellitus, Type 2/complications (1046). Other terms and subheadings also exhibited extensive connections within this SNA network, with Diabetic Nephropathies/prevention & control, Diabetic Nephropathies/pathology, Diabetic Nephropathies/diagnosis, Diabetic Nephropathies/metabolism, Diabetic Nephropathies/genetics, and Diabetes Mellitus, Type 2/drug therapy all exhibiting a high level of betweenness centrality consistent with their importance in the overall complex network. The average betweenness centrality in this analysis was 11.6363 ± 11.012 (Table 4). Newly emerging high-frequency MeSH terms/MeSH subheadings were represented by red squares in this analysis (Figure 4) and included Renal Insufficiency, Chronic/drug therapy, Albuminuria/drug therapy, Plant Extracts/pharmacology, Kidney Failure, Chronic/therapy, Mesangial Cells/metabolism, Polymorphism, Single Nucleotide, Diabetes Mellitus, Diabetes Mellitus and Type 2, and Diabetic Nephropathies.

## 4. Discussion

As global rates of T2DM continue to rise, so too does the incidence of diabetic kidney disease. As the onset of DN is often asymptomatic in its early stages, many diabetic patients exhibit proteinuria and, in some cases, nephrotic syndrome [13], with a subset of these patients ultimately developing ESRD and requiring dialysis. DN thus imposes a major medical and economic burden on affected patients, their families, and society as a whole [5]. However, research efforts pertaining to DN are complex and multifaceted, necessitating a comprehensive systematic overview of this research space in order to effectively identify current trends and emerging hotspots in the field. Here, we obtained 5,337 DN-related studies published in the PubMed database within the last 5 years and thereby explored the overall structure of this field and associated research progress pertaining to DN from 2016 to 2020, with promising research hotspots being identified through co-word, biclustering, strategic diagram, and SNA analyses.

Our analyses revealed that the majority of DN-related studies published during this period were from the USA (33.37%), followed by the UK (25.65%), the Netherlands (6.97%), and Germany (5.27%). The journals that published the largest proportion of the studies in this analysis included the *Journal of Diabetes Research* (151), *Scientific Reports* (150), *Journal of Diabetes and Its Complications* (146), *Kidney International* (107), *PLOS ONE* (95), *American Journal of Physiology-Renal Physiology* (95), and *Diabetes Care* (93). These journals are thus likely to continue publishing important discoveries in the DN research space in the near future.

Through a biclustering analysis, we grouped high-frequency MeSH terms/MeSH subheadings were aggregated into 5 clusters that were placed within a strategic diagram based on the results of strategic coordinate calculations.

The primary keywords included within cluster 0 were associated with the etiology, diagnosis, and blood/urinary biomarkers of DN. The etiological basis for DN has been an area of extensive research interest in recent years, revealing this condition to be associated with inflammatory response, glomerulosclerosis, renal tubular fibrosis, podocyte damage and loss, oxidative stress, autophagy, and apoptotic cell death [14–17]. There is also evidence that mitochondrial dysfunction and endoplasmic reticulum stress can contribute to the development of this condition [18, 19]. Measurements of urinary protein levels are commonly used for the diagnosis of DN in clinical practice, but other urinary and blood-based biomarkers have also been suggested to be of value for the early diagnosis and/or prognostic evaluation of DN patients [1, 20]. These biomarkers include fibrosis-related factors, exosomes, noncoding RNAs, secreted proteins, and proinflammatory cytokines [21, 22]. In the strategic diagram, cluster 0 was located within quadrant I, consistent with the central position and mature status of these topics, indicating that they have been active research hotspots over the past 5 years.

The primary keywords included in cluster 1 were associated with the drug-based treatment of DN and included antioxidant-based drugs, drugs targeting signaling pathways, plant extract drugs, and certain hypoglycemic drugs. In one recent report, p-coumaric acid was shown to decrease oxidative stress and thereby lower levels of glomerular hypertrophy and serum urea, creatinine, and uric acid levels in diabetic model rats [23]. The plant extract *Cissus quadrangularis* has been reported to improve DN-related outcomes owing to its ability to reverse hyperglycemia-induced SIRT1 downregulation [24], while *Camellia sinensis* L. can target the SIRT1/AMPK signaling axis to alleviate inflammation and reduce the severity of renal injury in diabetic model mice [25]. SGLT-2 inhibitors and glucagon-like peptide-1 receptor (GLP-1R) activators are hypoglycemic drugs with demonstrated renoprotective activity [6], and SGLT-2 inhibitors can also significantly reduce urinary protein levels in the context of DN. Cluster 1 was located within quadrant II in the generated strategic diagram, consistent with a high degree of research maturity but a lower core degree value. This may indicate that these studies have not been a primary focus of DN-related research over the past 5 years. Even so, as these analyses are directly related to DN patient prognosis and they are relatively mature, they may serve as core areas for future study.

Cluster 2 contained keywords pertaining to single nucleotide polymorphisms (SNPs) and the roles of glomerular mesangial cells and podocytes in DN. SNPs are heritable variations within the genome that have been best studied in the context of DN associated with type 1 diabetes mellitus, as it is a common complication in this patient population associated with higher mortality rates associated with early cardiovascular events [26]. The advent of genome-wide association studies has enabled more in-depth analyses of the relationships between SNPs and DN [27]. Glomerular mesangial cell proliferation and extracellular matrix deposition are two of the most prominent pathological changes observed in the context of DN. Hyperglycemic conditions can induce DN development by influencing glomerular mesangial cells through signaling mediated by a variety of inflammatory factors, cytokines, and associated signal transduction pathways [28]. Podocytes are a class of glomerular epithelial cells that form a slit membrane that functions as a barrier against proteinuria incidence. As such, podocyte dysfunction is closely tied to the incidence of proteinuria in DN patients. Given that podocytes are terminally differentiated and unable to divide or regenerate, the injury and loss of these cells is a key determinant of renal prognosis [17, 29]. Single-cell technology development has enabled further in-depth analyses of the etiology of DN at the single-cell level [30]. Cluster 2 was located within quadrant III in the generated strategic diagram, with consistent lower levels of maturity and a lack of a core position within the DN-related research space over the past 5 years, underscoring a need for further research. As studies of the genetic and epigenetic basis of DN continue to emerge, the topics included within this cluster may become more prominent, and associated research interest may thus continue to grow.

Cluster 3 encompassed keywords pertaining to the treatment, epidemiology, and pathophysiology of DN. In addition to the traditional treatment methods such as antioxidant treatment and the improvement of microcirculatory activity, treatment strategies utilizing SGLT-2 inhibitors, GLP-1R agonists, and ACEI/ARB drugs are also emerging as promising approaches to the treatment of DN [31]. The emergence of so-called “big data” analyses has enabled more robust research regarding the epidemiology of DN. Through analyses of multiple patient populations and databases, researchers can better clarify DN patient mortality rates, ESRD incidence in an effort to identify risk factors associated with these outcomes [32]. Further epidemiological analyses have the potential to guide the development and validation of prognostic models capable of gauging DN patient outcomes in a clinical setting to guide treatment efforts [33]. There have been many studies of the pathophysiology of DN at the biopsy, cellular, molecular, and single-cell molecular levels [34], with a variety of research techniques having been employed to clarify the drivers of this condition in an effort to develop more reliable preventative, diagnostic, and treatment strategies for affected patients.

Cluster 4 included terms pertaining to DN-related miRNAs and signal transduction pathways. Several studies have highlighted links between specific miRNAs and the pathogenesis, early diagnosis, and treatment of DN [7, 35, 36]. Moreover, there is evidence that exosomes and associated miRNAs can impact renal tissues either directly or in synergy with other factors to shape the development and progression of DN [37, 38]. Signal transduction pathways and associated regulatory networks are important areas of DN-related research that offer important insight into the etiological basis for this debilitating condition [39–41]. The development of drugs targeting DN-related signal transduction pathways may also hold great promise as an area for future research [42, 43]. Moreover, miRNAs and signal transduction pathways can interact with one another to form DN signal networks [44].

Both cluster 3 and cluster 4 were located within quadrant IV in the generated strategic diagram, suggesting that both are of relatively high importance in the DN research field given their high core degree values. However, the maturity of both clusters was relatively low, suggesting that further research pertaining to these topics is warranted and that they may represent promising hotspots for future research.

By combining a co-word analysis with an SNA, we sought to clarify the network structure of DN-related research over the past 5 years. These analyses revealed that the top three MeSH terms/MeSH subheadings with the highest co-occurrence frequency also exhibited the highest degree centrality. The degree centrality value was the highest for Diabetic Nephropathies/drug therapy, consistent with a high number of nodes being connected to these terms suggesting that drug-related DN treatment serves as an important bridge within the overall network. Betweenness centrality is the most robust index for evaluating individual terms within an SNA. As the node with the highest betweenness centrality was Diabetic Nephropathies/etiology, this suggests that this node is central within the overall network and that the etiology of DN may thus be central to most or all studies in this research space. Other terms with high levels of betweenness centrality included Diabetic Nephropathies/drug therapy, Diabetes Mellitus, Type 2/complications, Diabetic Nephropathies/prevention & control, and Diabetic Nephropathies/pathology, suggesting that these terms also occupy prominent positions within the DN research space. Other nodes were also identified as having emerged in recent years, including Polymorphism, Single Nucleotide, Kidney Failure, Chronic/therapy, Mesangial Cells/metabolism, Renal Insufficiency, Chronic/drug therapy, Plant Extracts/pharmacology, Albuminuria/drug therapy, Diabetes Mellitus, Diabetic Nephropathies, and Diabetes Mellitus, Type 2, all of which exhibited high levels of activity within this network. These results, together with the results of the above biclustering and strategic diagram analyses, suggest that the high-frequency MeSH terms/MeSH subheadings corresponding to several of these nodes are likely to correspond to future research hotspots.

## 5. Implications

In this paper, a variety of bibliometrics tools were applied to analyze relevant studies in the DN field included in the PubMed database from 2016 to 2020. Specifically, we conducted co-word, biclustering, strategic diagram, and SNA analyses based upon high-frequency MeSH terms and MeSH subheadings. We further sought to visualize results and knowledge structure within this research area. This work provides readers with a scientific and comprehensive view of the structure of knowledge in this field. In particular, our visual results give readers an accessible and intuitive impression of the status of DN-related research. In addition, through our analysis, future research hotspots and directions in the field of DN research can be predicted, providing a theoretical basis for subsequent scientific exploration.

## 6. Limitations

This study is subject to certain limitations. For one, we specifically focused on English language studies in the PubMed database, and studies in other languages were thus omitted from our analyses, potentially impacting our results. Second, only high-frequency MeSH terms and subheadings were selected for biclustering, strategic diagram, and SNA analyses. As such, relatively new MeSH terms and subheadings may have been excluded owing to their lower frequency, thus influencing our overall study results. Future analyses should thus employ multiple databases and algorithms in order to conduct more comprehensive analyses. Third, only the PubMed database was retrieved in this study, whereas the WoS database was not, resulting in the exclusion of some studies from this analysis. Fourth, this study only discussed research pertaining to DN published with the last 5 years.

## 7. Conclusions

In summary, we herein conducted a thorough review of DN-related studies published in PubMed over the last 5 years through a series of bibliometric analyses. Overall, we found that the etiology, pathology, diagnosis, prevention, and drug-based treatment of DN were the primary areas of research focus from 2016 to 2020, with future research thus being based on or associated with these topics. Moreover, we identified miRNAs, signal transduction pathways, SNPs, the roles of mesangial cells and podocytes in DN, and the use of plant extracts and hypoglycemic drugs for the treatment DN as emerging research hotspots over the past 5 years. Future clinical research should thus focus on these emerging hotspots.

## Figures and Tables

**Table 1:** Characteristics of DN-related studies in PubMed (2016-2020).

Rank		Publications,			Publications,	
	Country	*n* (%)		Top journal	*n* (%)	
1	United States	1781	(33.37)	*Journal of Diabetes Research*	151	(2.82)
2	England	1369	(25.65)	*Scientific Reports*	150	(2.80)
3	Netherlands	372	(6.97)	*Journal of Diabetes and Its Complications*	146	(2.73)
4	Germany	281	(5.27)	*Kidney International*	107	(2.00)
5	Switzerland	266	(4.98)	*PLOS One*	95	(1.77)
6	Japan	174	(3.26)	*American Journal of Physiology-Renal Physiology*	95	(1.77)
7	Ireland	119	(2.23)	*Diabetes Care*	93	(1.74)
8	Italy	112	(2.10)	*Journal of the American Society of Nephrology*	78	(1.46)
9	Australia	110	(2.06)	*Diabetes, Obesity & Metabolism*	72	(1.34)
10	France	100	(1.87)	*Diabetes*	69	(1.29)
11	China	94	(1.76)	*Diabetes Research and Clinical Practice*	63	(1.18)
12	Greece	63	(1.18)	*Biomedicine & Pharmacotherapy*	60	(1.12)
13	United Arab Emirates	52	(0.97)	*International Journal of Molecular Sciences*	59	(1.10)
14	Spain	49	(0.92)	*Diabetes & Metabolic Syndrome*	56	(1.05)
15	New Zealand	44	(0.82)	*Medicine*	55	(1.03)
16	Saudi Arabia	40	(0.75)	*Nephrol Dial Transplant*	54	(1.01)
17	Korea (South)	36	(0.67)	*Diabetologia*	52	(0.97)
18	India	35	(0.66)	*Life Sciences*	51	(0.95)
19	Brazil	33	(0.62)	*Renal Failure*	49	(0.92)
20	Iran	20	(0.37)	*Biochemical and Biophysical Research Communications*	48	(0.90)
Total		5150	(96.50)		1603	(29.94)

**Table 2:** Cluster analysis of high-frequency DN-related MeSH terms/MeSH subheadings.

Cluster	Number of MeSH term/MeSH subheadings		Cluster analysis
0	3,9,19,42,50,7,54,46,25,12,20	1.	Etiology of DN
		2.	Diagnosis of DN
		3.	Blood and urinary markers of DN
1	39,29,15,1,44,52,37,14,5,18,6,31,40	1.	Drugs treat DN by signal transduction or oxidative stress
		2.	Some plants extract drugs to treat DN
		3.	Hypoglycemic drugs for DN
2	47,16,38,21,53,32,4,35,55	1.	Single gene polymorphism of DN
		2.	MCs in DN pathology
		3.	Podocytes in DN pathology
3	13,48,17,43,10,27,30,8,26,41	1.	Treatment of DN
		2.	Epidemiology of the DN
		3.	Pathophysiology of the DN
4	51,24,36,11,22,23,28,2,33,49,34,45	1.	MicroRNAs in DN
		2.	Signal transduction in DN

**Table 3:** Individual centrality of DN (2016-2020).

Rank	Major MeSH terms/MeSH subheadings	Degree	Betweenness	Closeness	Rank	Major MeSH terms/MeSH subheadings	Degree	Betweenness	Closeness
1	**Diabetic Nephropathies/drug therapy**	1167	35.64902878	61	29	**Oxidative Stress/drug effects**	206	7.044936657	79
2	**Diabetic Nephropathies/metabolism**	898	27.9265995	63	30	**Diabetes Mellitus, Type 2/physiopathology**	160	8.698302269	76
3	**Diabetes Mellitus, Type 2/complications**	1046	35.63362122	61	31	**Sodium-Glucose Transporter 2 Inhibitors/therapeutic use**	248	6.357406139	82
4	**Diabetic Nephropathies/genetics**	458	26.97906876	64	32	**Diabetes Mellitus, Type 2 / genetics**	142	3.724227667	86
5	**Diabetic Nephropathies/prevention & control**	626	35.073246	60	33	**MicroRNAs/metabolism**	166	6.197879791	79
6	**Diabetes Mellitus, Type 2/drug therapy**	714	26.34806252	66	34	**Diabetes Mellitus, Type 2/metabolism**	174	16.12250137	69
7	**Diabetic Nephropathies/diagnosis**	562	29.09814835	64	35	**MicroRNAs/genetics**	128	6.311041355	82
8	**Diabetic Nephropathies/pathology**	466	33.63822937	60	36	**Podocytes/pathology**	146	3.63256979	82
9	**Diabetic Nephropathies/etiology**	491	46.17017365	57	37	**Hypoglycemic Agents/pharmacology**	170	6.250207901	82
10	**Diabetic Nephropathies/epidemiology**	393	11.70644283	74	38	**Diabetes Mellitus**	99	3.609862089	92
11	**Diabetic Nephropathies/physiopathology**	334	22.46341896	64	39	**Signal Transduction/drug effects**	133	2.63280654	83
12	**Diabetic Nephropathies/blood**	325	14.88596535	71	40	**Renal Insufficiency, Chronic/drug therapy**	154	1.707116842	93
13	**Diabetic Nephropathies/therapy**	199	16.03588295	71	41	**Glomerular Filtration Rate**	164	6.622590065	79
14	**Kidney/drug effects**	476	11.09681988	76	42	**Biomarkers/blood**	126	2.42141366	83
15	**Diabetes Mellitus, Experimental/drug therapy**	401	5.41108036	80	43	**Renal Insufficiency, Chronic/epidemiology**	117	5.463775158	85
16	**Diabetic Nephropathies**	100	3.054833651	96	44	**Albuminuria/drug therapy**	137	3.159614563	88
17	**Diabetic Nephropathies/complications**	161	10.9369669	74	45	**Podocytes/drug effects**	90	2.380554914	83
18	**Hypoglycemic Agents/therapeutic use**	391	14.7321043	70	46	**Diabetes Mellitus, Type 2/urine**	121	6.479604244	77
19	**Diabetes Mellitus, Type 1/complications**	269	11.97052002	77	47	**Diabetes Mellitus, Type 2**	63	1.609865546	96
20	**Diabetes Mellitus, Type 2/blood**	279	9.558413506	75	48	**Kidney Failure, Chronic/therapy**	74	0.748316169	93
21	**Diabetes Mellitus, Experimental/complications**	270	12.85853577	75	49	**Signal Transduction**	105	2.201956034	85
22	**Kidney/pathology**	278	20.10590553	67	50	**Diabetes Mellitus, Type 2/diagnosis**	136	1.499567509	84
23	**Kidney/metabolism**	256	14.14010525	71	51	**Glucose/metabolism**	87	5.031882763	79
24	**Podocytes/metabolism**	228	9.336707115	79	52	**Plant Extracts/pharmacology**	129	1.895391941	88
25	**Diabetic Nephropathies/urine**	178	9.577069283	74	53	**Polymorphism, Single Nucleotide**	90	0.402818322	96
26	**Kidney/physiopathology**	264	14.80787563	70	54	**Albuminuria/diagnosis**	133	3.04097867	83
27	**Diabetes Mellitus, Type 2/epidemiology**	249	8.595939636	76	55	**Mesangial Cells/metabolism**	85	0.789119542	90
28	**Diabetes Mellitus, Experimental/metabolism**	238	6.172931671	80					

**Table 4:** Descriptive statistics regarding DN-related centrality.

Centralization	Min.	Max.	Mean ± SD
Degree	63	1167	278.182 ± 237.135
Betweenness	0.403	46.17	11.6363 ± 11.012
Closeness	57	96	77.273 ± 10.082

## Data Availability

The author confirm that the data supporting all the results in this study are available in the article.
